# Prolonged Flow-Controlled Ventilation in a Patient With ARDS and Multiple Trauma: A Case Report

**DOI:** 10.1016/j.chest.2025.06.048

**Published:** 2025-12-09

**Authors:** Romana Erblich, Wolfgang Puchner, Matthias Noitz, Marius Knöll, Bernhard Eichler, Stephan Kalb, Dominik Jenny, Thomas Tschoellitsch, Jens Meier, Martin W. Dünser

**Affiliations:** a Department of Anaesthesiology and Critical Care Medicine, Kepler University Hospital and Johannes Kepler University, Linz, Austria

**Keywords:** ARDS, flow-controlled ventilation, trauma

## Abstract

Flow-controlled ventilation (FCV) is characterized by a bidirectional linearized gas flow translating into a constant flow. We report the prolonged use of FCV in a 30-year-old patient with major trauma, including severe traumatic brain injury and posttraumatic ARDS, because the patient sustained other severe injuries such as those to the spine and pelvis. Conventional mechanical ventilation failed to attain normoxia and normocapnia, leading to hemodynamic compromise and refractory intracranial hypertension. FCV was used as an off-label rescue therapy because prone positioning and extracorporeal membrane oxygenation were contraindicated. Within a few hours, ventilation improved despite lower minute volumes. This was paralleled by a reduction in norepinephrine requirements and normalization of intracranial pressure. FCV was continued for 96 hours. This case report underlines the potential benefits of FCV as a novel ventilation mode in patients with ARDS and justifies future studies evaluating the outcome effects of FCV in this complex population.

Flow-controlled ventilation (FCV) is characterized by a bidirectional linearized gas flow translating into a constant flow during inspiration and expiration. Differences between FCV and conventional mechanical ventilation include no pause phases during the respiratory cycle and direct measurement of airway pressures in the trachea.^1^^,^^2^ FCV is used commonly for intraoperative ventilation when small-bore endotracheal tubes are required.^3^ In these settings, FCV allows for adequate ventilation with minute volumes and driving pressures in the normal range.^3^ Compared with volume-controlled ventilation, FVC improves lung aeration and respiratory system compliance.^4^, ^5^, ^6^ In 2 porcine ARDS models, FCV improved oxygenation and CO_2_ removal with lower minute volumes and similar inspiratory pressures than volume-controlled or pressure-controlled ventilation.^7^^,^^8^ FCV was associated with an increase in normally aerated lung volumes, fewer signs of ventilator-induced lung injury,^7^ and faster hemodynamic stabilization.^8^ To date, only 2 small studies have evaluated the short-term (30 min and 4 hours) effects of FCV in patients with COVID-19-associated ARDS. During FCV, oxygenation and ventilation remained stable despite lower respiratory rates, peak inspiratory pressures, and minute volumes than with conventional mechanical ventilation.^9^^,^^10^ Herein, we report the first so far published, to our knowledge, clinical use of FCV in a patient with ARDS for > 24 hours.

## Case Report

**Figure 1:**
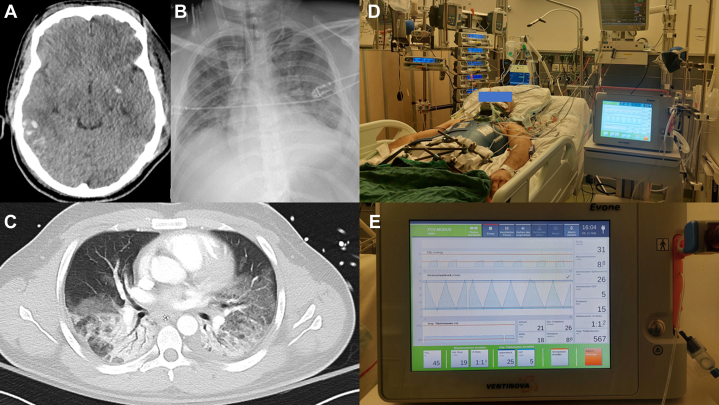
A-E, Imaging from the 30-year-old White male patient. A, Cerebral CT scan exhibiting brain edema, subarachnoid, and intracerebral hemorrhages. B, C, Chest radiograph (B) and CT scan (C) with features of ARDS. D, Photograph showing ICU setup. E, Photograph showing and flow-controlled ventilator (Evone; Ventinova Medical).

A 30-year-old White man (height, 170 cm; weight, 80 kg) was admitted with severe injuries: traumatic brain injury (Glasgow Coma Scale score, 4 points; multiple contusions and intracerebral and subarachnoid hemorrhages) (Fig 1A), unstable cervical spine fractures, bilateral rib fractures, bilateral lung contusions, subcapsular splenic hematoma, renal contusion, unstable pelvic ring fracture, and lower extremity fractures. The injury severity score was 59 points. After resuscitation, external fixation of the pelvis was performed emergently and an intracranial pressure (ICP) monitoring device was introduced. Despite cardiorespiratory stabilization, the absence of an intracranial hematoma amenable to surgical evacuation, deep multimodal sedation, osmotherapy, and normothermia, ICP peaked up to 25 mm Hg. Forty-eight hours after trauma, right-sided decompressive hemicraniectomy was performed. Simultaneously, a progressive deterioration in respiratory function required higher ventilator support with increasing inspiratory oxygen concentrations and minute volumes to attain normoxia and normocapnia. On day 3, ARDS had developed (Figs 1B, 1C, Table 1).^11^ Within a few hours, Paco_2_ rose to 68 mm Hg, triggering ICP plateaus of up to 26 mm Hg. Norepinephrine requirements had increased to 0.46 μg/kg/min to maintain cerebral perfusion pressure at 55 to 60 mm Hg. Prone positioning was contraindicated because of the unstable cervical spine injury and intracranial hypertension, as was venovenous extracorporeal membrane oxygenation because of intracranial hemorrhages and an increased overall bleeding risk. As a rescue therapy, mechanical ventilation was switched from pressure-controlled ventilation (Hamilton C6; Hamilton Medical) to FCV using a dedicated ventilator (Evone; Ventinova Medical) (Figs 1D, 1E). The endotracheal tube (inner diameter, 8.0 mm) was connected to the ventilator system using the conventional tube adapter (Evone Conventional Tube Adapter; Ventinova Medical), and the pressure monitoring catheter was introduced up to the tip of the endotracheal tube. As suggested in a previous study,^12^ FCV settings were individualized by compliance-guided positive end-expiratory pressure and peak pressure titration. Within 3 hours, the partial arterial oxygen and Paco_2_ improved, despite lower minute volumes (Table 1). Norepinephrine requirements decreased and ICP consistently fell to < 23 mm Hg. FCV was continued for 96 hours until ventilator lung function and ICP had stabilized, although this exceeded the maximum approved time for use of the FCV ventilator (< 72 hours). The patient then was switched back to pressure-controlled ventilation using a standard ventilator (Hamilton C6). Definitive surgical procedures (cervical spine stabilization, pelvic ring osteosynthesis) and tracheostomy were performed subsequently. The ensuing hospitalization was complicated by peripheral pulmonary embolism, nosocomial COVID-19, rhabdomyolysis-induced acute kidney injury, and ventilator-associated pneumonia. On day 31 after sustaining trauma, the patient was transferred from the ICU to the rehabilitation unit. At discharge, he was awake, was able to communicate, and exhibited a mild right-sided hemiparesis.

**Table 1:** Course of Ventilator Settings, Gas Exchange, and Other Clinical Parameters Before and During FCV

Variable	PCV	FCV Time Point (h)					
		3	12	24	48	72	96
Ventilation settings							
Inspiratory flow, L/min	65	19	19	19	20	19	20
Fio_2_	0.7	0.4	0.4	0.55	0.65	0.55	0.6
PEEP, mbar	12	8	5	7	8	8	8
Peak pressure, mbar	28	30	27	30	33	30	30
Tidal volume, mL	430	550	605	560	640	630	680
Compliance, mL/mbar	27	26	26	25	23	25	27
Resistance, mbar/L/s	8	9	11	9	NA	NA	NA
Respiratory rate, beats/min	25	15	14	15	14	14	13
Minute volume, L/min	10.8	8.6	8.8	8.9	8.9	8.8	8.9
Mechanical power, J/min^a^	21	16	16	16	19	18	18
Gas exchange							
Pao_2_ to Fio_2_ ratio	114	191	195	127	116	152	148
Paco_2_, mm Hg	68	44	40	46	46	45	44
pH	7.26	7.39	7.41	7.35	7.36	7.42	7.47
Other clinical parameters							
ICP, mm Hg	24	22	22	20	18	14	12
Heart rate, beats/min	101	107	93	94	90	87	96
Mean arterial pressure, mm Hg	91	74	85	69	89	77	97
Norepinephrine dose, μg/kg/min	0.46	0.31	0.32	0.39	0.33	0.19	0

FCV = flow-controlled ventilation; ICP = intracranial pressure; NA = not available; PCV = pressure-controlled ventilation; PEEP = positive end-expiratory pressure.

a Calculated according to the following formula: 0.098 × respiratory rate × tidal volume (L) × (peak pressure – 0.5 × [plateau pressure – PEEP]).^11^

## Discussion

In this patient with ARDS, FCV was used as an off-label rescue therapy over 96 hours and delivered through a standard size endotracheal tube, because both prone positioning and extracorporeal membrane oxygenation were considered contraindicated. Within a short period of FCV, we observed a substantial improvement in ventilation and CO_2_ elimination, possibly resulting from better lung aeration and reduced dead space.^4^, ^5^, ^6^ Consecutive improvements in ICP and hemodynamic function paralleled the reversal of hypercapnia. Interestingly, oxygenation improved only moderately during FCV, whereas lung compliance remained unchanged. Although peak inspiratory pressures during FCV were higher than during pressure-controlled ventilation, mean airway pressures were probably lower, given that both peak and end-expiratory pressures during FCV are maintained only for very short moments during the respiratory cycle. Accordingly, the mechanical power delivered during FCV in this patient was approximately 25% lower than during conventional pressure-controlled ventilation. This finding is in line with results of prior studies.^10^

FCV also was associated with disadvantages in this patient. First, endotracheal suctioning was possible only when the ventilator circuit was disconnected and the pressure monitoring catheter was removed partially. Second, the patient required the maximum inspiratory flow rate that the FCV ventilator was able to deliver to achieve a sufficient minute ventilation. No safety margin was left. Furthermore, operation of the FCV ventilator resulted in a relevant noise level perceived as disturbing by other patients and staff. Although not immediately pertinent to this patient, who required deep sedation because of intracranial hypertension, the FCV ventilator would have not allowed for spontaneous or assisted ventilation.

This case report underlines the potential benefits of FCV as a novel ventilation method in patients with ARDS and justifies future studies evaluating the outcome effects of FCV in this population with complex injuries.
